# Isodeoxyelephantopin Inactivates Thioredoxin Reductase 1 and Activates ROS-Mediated JNK Signaling Pathway to Exacerbate Cisplatin Effectiveness in Human Colon Cancer Cells

**DOI:** 10.3389/fcell.2020.580517

**Published:** 2020-09-22

**Authors:** Lin Hong, Jundixia Chen, Fang Wu, Fengjiao Wu, Xin Shen, Peisen Zheng, Rongrong Shao, Kongqin Lu, Zhiguo Liu, Daoxing Chen, Guang Liang, Yuepiao Cai, Peng Zou, Yiqun Xia

**Affiliations:** ^1^The First Affiliated Hospital of Wenzhou Medical University, Wenzhou Medical University, Wenzhou, China; ^2^Cancer and Anticancer Drug Research Center, School of Pharmaceutical Sciences, Wenzhou Medical University, Wenzhou, China; ^3^Zhuji Institute of Biomedicine, School of Pharmaceutical Sciences, Wenzhou Medical University, Zhuji, China; ^4^Wenzhou University-Wenzhou Medical University Collaborative Innovation Center of Biomedical, Wenzhou, China

**Keywords:** isodeoxyelephantopin, oxidative stress, thioredoxin reductase 1, cisplatin, JNK

## Abstract

Colon cancer is one of the leading causes of cancer-related death in the world. The development of new drugs and therapeutic strategies for patients with colon cancer are urgently needed. Isodeoxyelephantopin (ESI), a sesquiterpene lactone isolated from the medicinal plant *Elephantopus scaber* L., has been reported to exert antitumor effects on several cancer cells. However, the molecular mechanisms underlying the action of ESI is still elusive. In the present study, we found that ESI potently suppressed cell proliferation in human colon cancer cells. Furthermore, our results showed that ESI treatment markedly increased cellular reactive oxygen species (ROS) levels by inhibiting thioredoxin reductase 1 (TrxR1) activity, which leads to activation of the JNK signaling pathway and eventually cell death in HCT116 and RKO cells. Importantly, we found that ESI markedly enhanced cisplatin-induced cytotoxicity in HCT116 and RKO cells. Combination of ESI and cisplatin significantly increased the production of ROS, resulting in activation of the JNK signaling pathway in HCT116 and RKO cells. *In vivo*, we found that ESI combined with cisplatin significantly suppressed tumor growth in HCT116 xenograft models. Together, our study provide a preclinical proof-of-concept for ESI as a potential strategy for colon cancer treatment.

## Introduction

Colon cancer is a significant public health problem and one of the leading causes of cancer-related death in the world. Despite advances in surgery, radiation therapy and chemotherapy, the overall survival rate of patients with colon cancer is still not optimistic (Arnold et al., 2017). Therefore, novel therapeutic strategies for patients with colon cancer are urgently needed. Natural products have been used for treatment or prevention of various human diseases for centuries, particularly in cancer therapy (Newman and Cragg, 2016). *Elephantopus scaber* L. is a traditional medicinal herb with multiple medicinal uses. In Chinese medicine, the extract of this plant is used as an antiviral, antidiuretic, and antibacterial agent as well as in the treatment of bronchitis, hepatitis, and arthralgia (Poli et al., 1992; Rajesh and Latha, 2001; Li et al., 2004). Isodeoxyelephantopin (ESI), a sesquiterpene lactone isolated from *Elephantopus scaber* L, has been reported to exert antitumor effects in several malignant carcinomas (Yan et al., 2013; Verma et al., 2019). A previous study demonstrated that ESI induces cell cycle arrest at G2/M phase in T47D cells (Kabeer et al., 2014). ESI was also found to inhibit the growth of human chronic myeloid leukemia cells by inhibiting NF-κB activation and NF-κB-regulated gene expression (Ichikawa et al., 2006). In lung cancer cells, ESI favored cell survival by activating protective autophagy (Wang et al., 2017). However, the antitumor effects of ESI on colon cancer has not been reported till now, and the molecular mechanisms underlying the action of ESI is still elusive.

Cisplatin is one of the most successful chemotherapeutics and has been widely used in clinics for the treatment of cancer (Wang and Lippard, 2005). The mechanism of action of cisplatin has been broadly studied in the past decades. It is generally agreed that DNA is a major target for cisplatin (Jung and Lippard, 2007; Basu and Krishnamurthy, 2010). Various signal transduction pathways and molecules, including p53, Nrf2, MAPK, and PD-L1, are involved in the process of cisplatin-induced cell death (Bragado et al., 2007; Fournel et al., 2019; Liao et al., 2019). However, many patients rapidly acquire resistance to cisplatin treatment during therapy, and the molecular mechanisms of cisplatin resistance remains enigmatic (Ahmed et al., 2018; Roy et al., 2018; Cruz-Bermudez et al., 2019; Su et al., 2019). It has been suggested that cisplatin in combination with other herb compounds is more effective than cisplatin alone (Wang J. et al., 2018; Wang Y. et al., 2018). Therefore, it is interesting to investigate the synergistic effect of cisplatin in combination with ESI for the treatment of colon cancer.

In this study, we investigated the molecular mechanisms underlying the action of ESI in human colon cancer cells. We observed that ESI significantly inhibited TrxR1 activity and increased the accumulation of ROS, which leads to activation of the JNK signaling pathway and eventually cell death in HCT116 and RKO cells. Importantly, we found that ESI significantly enhanced cisplatin-induced cytotoxicity in HCT116 and RKO cells. Moreover, ESI in combination with cisplatin markedly suppressed tumor growth in HCT116 xenograft models. Together, our data provide new insight into the mechanisms of antitumor action of ESI, and suggest that ESI might be a potential candidate for the treatment of colon cancer.

## Results

### ESI Treatment Increases ROS Levels in Human Colon Cancer Cells

**FIGURE 1:**
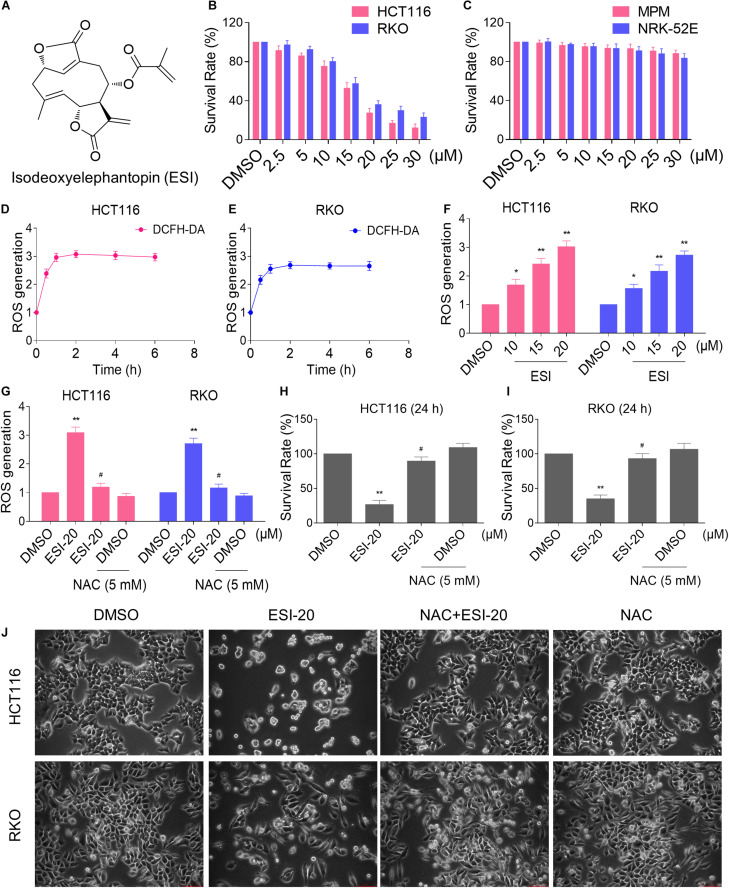
ESI inhibits cell proliferation and increases ROS levels in HCT116 and RKO cells. **(A)** Chemical structure of ESI. **(B)** Cell viability was measured in HCT116 and RKO cells after treated with ESI for 24 h. **(C)** Cell viability was measured in MPM and NRK-52E cells after treated with ESI for 24 h. **(D,E)** Intracellular ROS levels were measured in HCT116 and RKO cells after treated with ESI (20 μM) for indicated time periods. **(F)** Intracellular ROS levels were measured in HCT116 and RKO cells after treated with ESI for 2 h. **(G)** Cells were pretreated with NAC (5 mM) for 2 h before exposure to ESI. Intracellular ROS levels were measured after treated with ESI (20 μM) for 2 h. **(H,I)** Cells were pretreated with NAC (5 mM) for 2 h before exposure to ESI. Cell viability was measured after treated with ESI for 24 h. **(J)** Cells were pretreated with NAC (5 mM) for 2 h and cell morphology was observed after treated with ESI for 24 h. Data from three technical replicates (*p < 0.05, **p < 0.01 versus DMSO group, ^#^*p* < 0.05 versus ESI-20 group).

We first tested the cytotoxic effect of ESI (Figure 1A) on the viability of colon cancer cells and normal cells. As shown in Figures 1B,C, there were significant reductions in the viability of two colon cancer cell lines upon ESI treatment, but has little effect on normal MPM and NRK-52E cells. Next, we set out to investigate the molecular mechanisms underlying the action of ESI. Recent studies showed that ROS generation plays an important role in the antitumor action of some natural compounds (Dias et al., 2018; Liu et al., 2018). Therefore, we measured the intracellular ROS levels after ESI treatment. Time-course results showed that ESI treatment markedly induced ROS generation in HCT116 and RKO cells (Figures 1D,E). In addition, we found that treatment with ESI for 2 h caused a dose-dependent increase in ROS levels (Figure 1F). To determine the role of ROS in mediating the antitumor effect of ESI, the ROS scavenger NAC was used in our experiment. We found that pretreatment with NAC markedly reversed ESI-induced increase in ROS levels and cell death rate in HCT116 and RKO cells (Figures 1G–J). These data suggest that ROS generation plays an essential role in ESI-induced cytotoxicity in colon cancer cells.

### ESI Inactivates TrxR1 in Human Colon Cancer Cells

**FIGURE 2:**
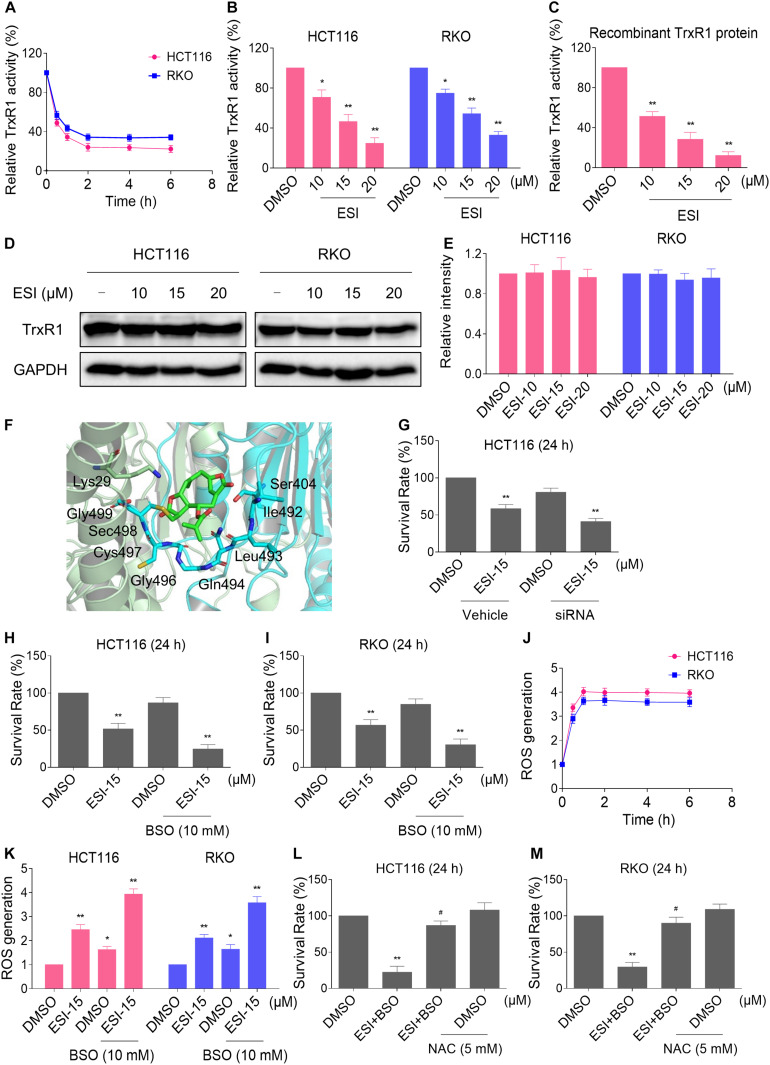
ESI inhibits TrxR1 activity in HCT116 and RKO cells. **(A)** TrxR1 activity was measured by the endpoint insulin reduction assay after treated with ESI (20 μM) for indicated time periods. **(B)** TrxR1 activity was measured by the endpoint insulin reduction assay after treated with ESI for 2 h. **(C)** TrxR1 protein activity was measured by the DTNB assay after treated with ESI for 2 h. **(D,E)** HCT116 and RKO cells were treated with ESI for 12 h and then lysed for Western blot analyses with the indicated antibodies. The intensities of TrxR1 and GAPDH bands were quantified using ImageJ software. TrxR1 protein levels were normalized to GAPDH. **(F)** Molecular docking of ESI with TrxR1 protein was carried out with the docking software. **(G)** HCT116 cells transfected with TrxR1 siRNA or control siRNA were treated with ESI for 24 h. Cell viability was measured using a methyl thiazolyl tetrazolium assay. **(H,I)** Cell viability was measured after treated with ESI or BSO alone or their combination for 24 h. **(J)** Intracellular ROS levels were measured after treated with ESI (15 μM) and BSO (10 mM) combination for indicated time periods. **(K)** Intracellular ROS levels were measured after treated with ESI or BSO alone or their combination for 2 h. **(L,M)** Cells were pretreated with NAC (5 mM) for 2 h and cell viability was measured after treated with ESI (15 μM) and BSO (10 mM) combination for 24 h. Data from three technical replicates (*p < 0.05, **p < 0.01 versus DMSO group, ^#^*p* < 0.05 versus ESI+BSO group).

Thioredoxin reductase 1 is a key regulator of cellular redox balance and accumulating evidence suggest that ROS accumulation may be increased when TrxR1 activity is inhibited (Duan et al., 2016; Dagnell et al., 2018; Zheng et al., 2019). Therefore, we tested the inhibitory effect of ESI on TrxR1 activity in colon cancer cells. Using an endpoint insulin reduction assay to quantify inhibition of TrxR1 activity, we found that ESI treatment inhibited the TrxR1 activity in a time- and dose-dependent manner in HCT116 and RKO cells (Figures 2A,B). Remarkably, we found that ESI directly inhibited the TrxR1 protein activity in a dose-dependent manner (Figure 2C). The densitometric analysis of Western blot bands showed that the expression level of TrxR1 did not significantly change after treated with ESI (Figures 2D,E). In addition, we performed a molecular simulation of ESI-TrxR1 complex using docking software. As shown in Figure 2F, the key residues around ESI included Gly499, Sec498, Cys497, Gly496, Gln494, Leu493, Ile492, Ser404 and Lys29. Thus, the proposed reaction mechanism for ESI is to block the adjacent C-terminal active site residues Cys and Sec of TrxR1, which is expected to effectively suppress TrxR1 activity (Xu et al., 2016). To further address the physiological relevance of TrxR1-mediated ESI cytotoxicity, we knocked down TrxR1 expression by using siRNA in HCT116 cells. The TrxR1 knockdown by siRNA resulted in an appreciable increase in ESI-induced cell death in HCT116 cells (Figure 2G).

Glutathione (GSH) is the most abundant antioxidant in cells, and plays a critical role in cellular antioxidant defenses. GSH acting in concert with its dependent enzymes, known as the GSH system, which is another redox regulatory network in cells besides the thioredoxin system, and it also acts as a backup of the thioredoxin system (Du et al., 2012; Harris et al., 2015). L-Buthionine-sulfoximine (BSO) is a sulfoximine which reduces levels of GSH and is being investigated as an adjunct with chemotherapy in the treatment of cancer (Lien et al., 2016; Rashmi et al., 2018). Therefore, we set out to evaluate the synergistic effects of ESI and BSO. Using the MTT assay, we found that ESI in combination with BSO exhibited a synergistic effect against both HCT116 and RKO cells (Figures 2H,I). Furthermore, compared with ESI or BSO treatment alone, the combined treatment greatly increased ROS levels in HCT116 and RKO cells (Figures 2J,K). To investigate the role of ROS in the combined treatment-induced cell death, the cells were treated with the combination of ESI and BSO after pretreated with antioxidant NAC. As shown in Figures 2L,M, NAC pretreatment significantly attenuated the combined treatment-induced cytotoxicity in both HCT116 and RKO cells. Taken together, these data indicate that ESI induces ROS-mediated cell death by inhibiting TrxR1 activity.

### ESI Activates JNK Signaling Pathway in Human Colon Cancer Cells

**FIGURE 3:**
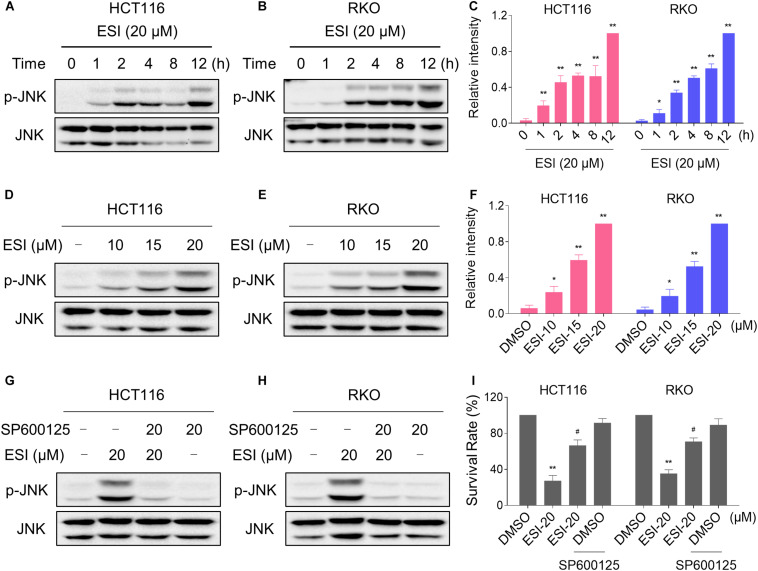
ESI activates JNK signaling pathway in HCT116 and RKO cells. **(A–C)** Cells were treated with ESI for indicated time periods and then lysed for Western blot analyses with the indicated antibodies. The intensities of p-JNK and JNK bands were quantified using ImageJ software. p-JNK protein levels were normalized to JNK. **(D–F)** Cells were treated with ESI for 12 h and then lysed for Western blot analyses with the indicated antibodies. **(G,H)** Cells were pretreated with SP600125 (20 μM) for 2 h before exposure to ESI. Cell lysates were blotted with the indicated antibodies after treated with ESI for 12 h. **(I)** Cells were pretreated with SP600125 (20 μM) for 2 h and cell viability was measured after treated with ESI for 24 h. Data from three technical replicates (*p < 0.05, **p < 0.01 versus DMSO group, ^#^*p* < 0.05 versus ESI-20 group).

In the presence of ROS, the oxidized thioredoxin (Trx) form is released and subsequently activates apoptosis signal-regulating kinase 1 to induce cell death via activation of the JNK signaling pathway (Jin et al., 2015; Mantzaris et al., 2016). Therefore, we set out to determine whether the JNK signaling pathway was activated in HCT116 and RKO cell lines when treated with ESI. As shown in Figures 3A–C, the JNK signaling pathway was indeed activated in both cell lines. In addition, ESI treatment increased the phosphorylation of JNK in a dose-dependently manner (Figures 3D–F). We next sought to determine the role of JNK signaling pathway in mediating ESI-induced cell death in HCT116 and RKO cells. As shown in Figures 3G,H, the phosphorylation of JNK induced by ESI was greatly reversed when pre-treated with SP600125 (a JNK inhibitor). This was associated with an appreciable reduction in ESI-induced cell death in HCT116 and RKO cells, indicating that JNK activation is essential for ESI-induced cell death in colon cancer cells (Figure 3I).

**FIGURE 4:**
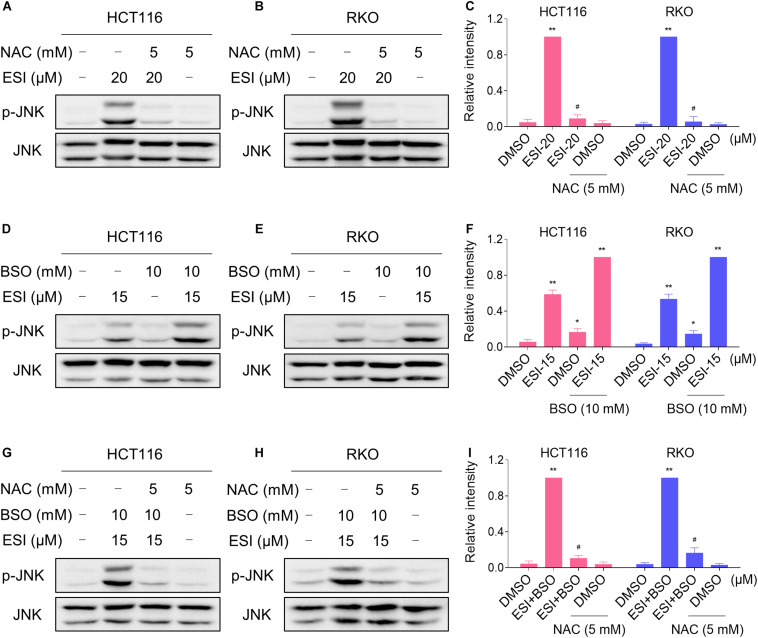
ESI activates ROS-dependent JNK signaling pathway in HCT116 and RKO cells. **(A–C)** Cells were pretreated with NAC (5 mM) for 2 h before exposure to ESI. Cell lysates were blotted with the indicated antibodies after treated with ESI for 12 h. **(D–F)** Cells were treated with ESI or BSO alone or their combination for 12 h and then lysed for Western blot analyses with the indicated antibodies. **(G–I)** Cells were pretreated with NAC (5 mM) for 2 h and cell lysates were blotted with the indicated antibodies after treated with ESI (15 μM) and BSO (10 mM) combination for 12 h. Data from three technical replicates (*p < 0.05, **p < 0.01 versus DMSO group, ^#^*p* < 0.05 versus ESI-20 or ESI+BSO group).

We next investigated the relationship between ROS generation and JNK activation in colon cancer cells. As shown in Figures 4A–C, the phosphorylation of JNK induced by ESI was significantly reversed when pre-treated with NAC. To further extend this observation, we measured the level of JNK phosphorylation in HCT116 and RKO cells after treated with ESI and BSO combination. As shown in Figures 4D–F, ESI and BSO synergistically increased the level of JNK phosphorylation in both cell lines. Moreover, the combined treatment-induced phosphorylation of JNK was markedly reversed by NAC pretreatment in both HCT116 and RKO cells (Figures 4G–I). Together, these findings indicate that the JNK signaling pathway is a downstream effector of ROS induced by the combined treatment in colon cancer cells.

### ESI and Cisplatin Combination Increases ROS Levels in Human Colon Cancer Cells

**FIGURE 5:**
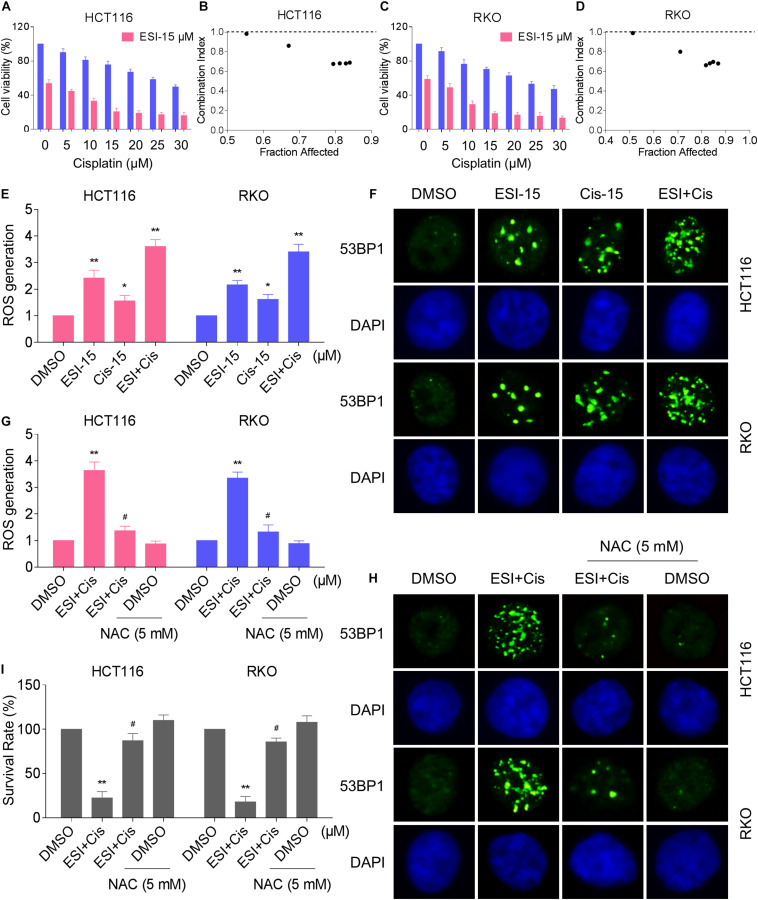
ESI and cisplatin combination increases ROS levels in HCT116 and RKO cells. **(A,C)** Cell viability was measured after treated with ESI or cisplatin alone or their combination for 24 h. **(B,D)** Combination index (CI) values were calculated from the MTT assays using Calcusyn software. **(E)** Intracellular ROS levels were measured after treated with ESI (15 μM) or cisplatin (15 μM) alone or their combination (15 μM ESI and 15 μM cisplatin) for 2 h. **(F)** The nuclear foci formation of 53BPl was detected after treated with ESI (15 μM) or cisplatin (15 μM) alone or their combination (15 μM ESI and 15 μM cisplatin) for 20 h. **(G)** Cells were pretreated with NAC (5 mM) for 2 h and intracellular ROS levels were measured after treated with ESI (15 μM) and cisplatin (15 μM) combination for 2 h. **(H)** Cells were pretreated with NAC (5 mM) for 2 h and nuclear foci formation of 53BPl was detected after treated with ESI (15 μM) and cisplatin (15 μM) combination for 20 h. **(I)** Cells were pretreated with NAC (5 mM) for 2 h and cell viability was measured after treated with ESI (15 μM) and cisplatin (15 μM) combination for 24 h. Data from three technical replicates (*p < 0.05, **p < 0.01 versus DMSO group, ^#^*p* < 0.05 versus ESI+Cis group).

Several studies showed that some ROS inducers can sensitize the tumor cells to cisplatin (Yang et al., 2017; Sun et al., 2018; Hsu et al., 2019; Zhang et al., 2019). Therefore, we set out to determine the synergistic effects of ESI and cisplatin. Using the MTT assay, we found that 15 μM ESI greatly increased the cytotoxicity of cisplatin in HCT116 and RKO cells (Figures 5A,C). The CI values were calculated from the MTT assay and suggested that ESI in combination with cisplatin exhibited a synergistic effect against both HCT116 and RKO cells (Figures 5B,D). Since ROS generation plays a critical role in ESI-induced cell death, we set out to determine whether ROS was upregulated in the HCT116 and RKO cell lines when treated with ESI and cisplatin combination. As shown in Figure 5E, ESI and cisplatin synergistically increased the levels of ROS in both cell lines.

Excessive amounts of ROS can cause oxidative damage to lipids and DNA (Park et al., 2018; Srinivas et al., 2018). Using an immunofluorescence assay, we found that combined treatment with ESI and cisplatin resulted in a significant accumulation of nuclear 53BP1 foci in HCT116 and RKO cells (Figure 5F). In addition, the combined treatment-induced accumulation of ROS and nuclear 53BP1 foci were markedly reversed by NAC pretreatment in both cell lines (Figures 5G,H). To further investigate the role of ROS in the combined treatment-induced cell death, the cells were treated with the combination of ESI and cisplatin after pre-treated with antioxidant NAC. As shown in Figure 5I, NAC pretreatment greatly attenuated the combined treatment-induced cytotoxicity in both HCT116 and RKO cells. Taken together, these data indicate that ESI and cisplatin combination induces ROS-mediated cell death in colon cancer cells.

### ESI and Cisplatin Cooperated to Activate ROS-Dependent JNK Signaling Pathway

**FIGURE 6:**
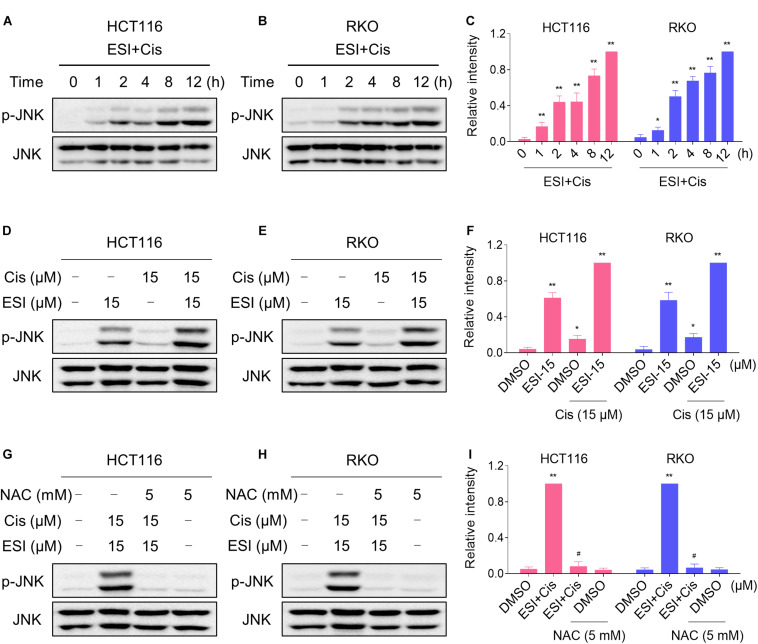
ESI and cisplatin cooperated to activate ROS-dependent JNK signaling pathway. **(A–C)** Cells were treated with ESI (15 μM) and cisplatin (15 μM) combination for indicated time periods and then lysed for Western blot analyses with the indicated antibodies. **(D–F)** Cells were treated with ESI or cisplatin alone or their combination for 12 h and then lysed for Western blot analyses with the indicated antibodies. **(G–I)** Cells were pretreated with NAC (5 mM) for 2 h and cell lysates were blotted with the indicated antibodies after treated with ESI (15 μM) and cisplatin (15 μM) combination for 12 h. Data from three technical replicates (*p < 0.05, **p < 0.01 versus DMSO group, ^#^*p* < 0.05 versus ESI+Cis group).

We next tested if the JNK signaling pathway was activated in HCT116 and RKO cell lines when treated with ESI and cisplatin. As shown in Figures 6A–C, ESI in combination with cisplatin increased the level of JNK phosphorylation in a time-dependently manner. Moreover, ESI and cisplatin synergistically increased the level of JNK phosphorylation in both cell lines (Figures 6D–F). We then attempted to investigate the relationship between ROS generation and JNK activation induced by the combined treatment in HCT116 and RKO cells. As shown in Figures 6G–I, the combined treatment-induced phosphorylation of JNK was markedly reversed by NAC pretreatment in both cell lines, indicating that activation of the JNK signaling pathway is due to accumulation of intracellular ROS in colon cancer cells.

### ESI and Cisplatin Cooperated to Inhibit Tumor Growth of HCT116 Xenografts in Nude Mice

**FIGURE 7:**
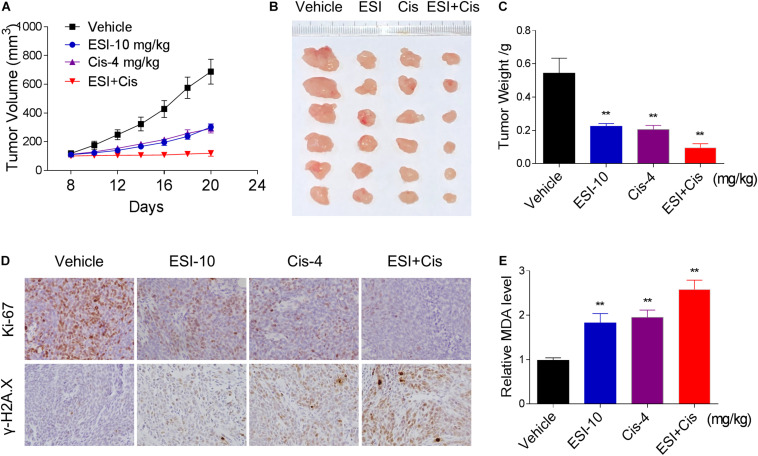
ESI and cisplatin cooperated to inhibit tumor growth of HCT116 xenografts in nude mice. **(A,B)** ESI (10 mg/kg) and cisplatin (4 mg/kg) combined treatment significantly decreased tumor volume and tumor weight **(C)** of HCT116 human colon cancer xenografts in nude mice. **(D)** The levels of Ki-67 and γH2A.X in tumor tissues. **(E)** MDA levels in tumor tissues (**p < 0.01 versus Vehicle group).

To extend our finding *in vivo*, we inoculated HCT116 cells into the athymic mice subcutaneously. The mice were equally divided into four groups (six mice/group) and received the following treatments: (1) control vehicle; (2) ESI (10 mg/kg); (3) cisplatin (4 mg/kg); (4) ESI (10 mg/kg) plus cisplatin (4 mg/kg). As shown in Figures 7A–C, 10 mg/kg ESI or 4 mg/kg cisplatin treatment effectively reduced tumor growth of HCT116 xenografts. Remarkably, the combined treatment with ESI and cisplatin showed stronger inhibitory effect on tumor growth in nude mice. Mechanistically, ESI and cisplatin synergistically inhibited the expression of Ki-67 and increased the level of γ-H2A.X in the tumor tissues (Figure 7D). Furthermore, we found that ESI in combination with cisplatin markedly increased the level of MDA, a marker of oxidative stress, in the tumor tissues (Figure 7E). These *in vivo* data support our findings in cell culture experiments and further strengthen the hypotheses that the generation of ROS is critical for the synergistic effect of ESI and cisplatin.

## Discussion

Colon cancer is one of the leading causes of cancer-related deaths worldwide. Chemotherapy remains an important therapeutic strategy for colon cancer. However, the application of conventional chemotherapeutic drugs is limited due to drug resistance and toxicities (Rabik and Dolan, 2007; Al-Batran et al., 2019; Pan et al., 2019). Therefore, the development of more effective drugs and/or drug combinations for colon cancer has high priority. Here, we investigated the effect and mechanism of ESI in colon cancer cells. We found that ESI potently inhibited the growth of colon cancer cells *in vitro* and in nude mice. Remarkably, we verified TrxR1 was a target of ESI and showed that ESI induced ROS generation by inhibiting TrxR1 activity. In addition, we showed that ESI has synergistic effects with the frontline chemotherapeutic agent cisplatin, suggesting that such a combinatorial treatment might be a more effective strategy for colon cancer treatment.

Under physiological conditions, ROS production and elimination is tightly regulated. Compared with normal cells, cancer cells usually generate and maintain higher ROS levels due to distorted metabolism (Glasauer and Chandel, 2014; Schieber and Chandel, 2014). Elevated ROS levels render cancer cells more sensitive to agents that increases ROS generation. Therefore, manipulating ROS levels by redox modulation is a useful strategy to selectively kill cancer cells (Trachootham et al., 2009; Gorrini et al., 2013). In the present study, we showed that ESI treatment resulted in a significant increase in intracellular ROS levels, and that pretreatment with NAC significantly reversed ESI-induced ROS generation and cell death, indicating that ROS play an important role in the antitumor activity of ESI. We also identified the downstream effector of ROS induced by ESI in the cell death process. We found that ESI treatment concomitantly activated the JNK signaling pathway, as indicated by increased phosphorylation of JNK. Moreover, we found that pretreatment with NAC markedly reversed ESI-induced phosphorylation of JNK in colon cancer cells, suggesting that ROS acts as an upstream signaling molecule involved in ESI-induced activation of the JNK signaling pathway.

Understanding the molecular mechanism underlying the antitumor action of ESI may optimize the design of ESI-based therapies. TrxR1 is a selenoprotein that functions to reduce the oxidoreductase Trx in a NADPH dependent manner, and plays a critical role in regulating the cellular redox balance (Arner, 2017). Accumulating evidence indicates that intracellular ROS levels may be increased when the TrxR1 activity is chemically inhibited (Duan et al., 2016; Dagnell et al., 2018; Zheng et al., 2019). Accordingly, we found that TrxR1 activity in colon cancer cells was decreased with increasing ESI concentration. In addition, we demonstrated that ESI directly inhibited the TrxR1 protein activity in a dose-dependent manner. The densitometric analysis of Western blot bands revealed that ESI treatment does not affect the expression of TrxR1 in colon cancer cells. Furthermore, we found that TrxR1 knockdown sensitized cells to ESI, which was consistent with previous studies (Duan et al., 2016; Yao et al., 2020). The thioredoxin and GSH systems play important roles in regulating the cellular redox balance (Du et al., 2012; Harris et al., 2015; Kengen et al., 2018). Interestingly, we found that BSO significantly enhanced ESI-induced cell death in HCT116 and RKO cells via promoting generation of ROS, indicating that a combination therapy inhibiting both thioredoxin and GSH systems may become an effective way to treat colon cancer. Further insight into the roles of other antioxidant systems such as Nrf2 and GPX4, and how they act both alone and together, will provide important clues into more effective therapies for cancer patients.

A number of physical treatments or antitumor drugs, such as exemestane (Nuvoli et al., 2018), sorafenib (Roh et al., 2017), cisplatin (Pan et al., 2019), osimertinib (Tang et al., 2017), and irradiation (He et al., 2015), act, at least in part, through the generation of ROS. In this study, we showed that ESI significantly enhanced cisplatin-induced cell death in HCT116 and RKO cells via promoting generation of ROS and activation of the JNK signaling pathway. In addition, we demonstrated that ESI enhanced DNA damage induced by cisplatin based on increased formation of nuclear 53BP1 foci. The observation that ESI sensitizes the response of colon cancer cells to cisplatin may provide a promising strategy for colon cancer treatment: combination of ESI with existing oxidative stress-causing antitumor drugs or physical treatments, such as ionizing radiation (IR) and photodynamic therapy (PDT).

In conclusion, we have discovered a novel small molecule inhibitor of TrxR1, and showed that ESI induced cell death through ROS-mediated JNK signaling pathway in colon cancer cells. Our findings clearly demonstrated that ESI can be developed as a novel anticancer drug for the treatment of colon cancer. Furthermore, we found that ESI significantly enhanced the antitumor activity of cisplatin *in vitro* and *in vivo*. These findings provided new insight into the molecular mechanisms of antitumor action of ESI, which may provide potential therapies for the treatment of colon cancer.

## Materials and Methods

### Materials

Isodeoxyelephantopin (ESI) was purchased from Chengdu Herbpurify Co., Ltd. (Chengdu, China). ESI was dissolved in dimethyl sulfoxide (DMSO). JNK inhibitor SP600125 was obtained from Selleck Chemicals (Houston, TX, United States). L-Buthionine-sulfoximine (BSO) was purchased from Aladdin Industrial Corporation (Shanghai, China). NAC was purchased from Sigma (St. Louis, MO, United States). Antibodies of p-JNK and JNK were purchased from Cell Signaling Technology (Danvers, MA, United States). Antibodies of TrxR1 and GAPDH were purchased from Santa Cruz Biotechnology (Santa Cruz, CA, United States). Antibodies of Ki-67 and γ-H2A.X antibody were purchased from Abcam (Cambridge, MA, United States). The 53BP1 antibody was purchased from Novus Biologicals (Littleton, CO, United States).

### Cell Culture

HCT116, RKO and NRK-52E cell lines were obtained from the Cell Bank of Shanghai Institute of Biochemistry and Cell Biology, Chinese Academy of Sciences. HCT116 cells were grown in McCoy’s 5A medium plus 10% fetal bovine serum (FBS). RKO cells were grown in minimum essential medium plus 10% FBS. NRK-52E cells were grown in DMEM plus 10% FBS. Mouse peritoneal macrophage (MPM) cells were obtained as previously described (Zhao et al., 2015). All the cells were cultured in a humidified incubator with 5% CO_2_ at 37°C.

### Cell Viability Assay

Approximately 8,000 cells per well were seeded in 96-well plates and incubated overnight. Next, the cells were treated with ESI or cisplatin alone or their combination for 24 h. Cell viability was measured using a methyl thiazolyl tetrazolium assay. The drug interaction was evaluated by using the CI according to the Chou-Talalay method (Chou, 2010).

### Measurement of Intracellular ROS

The fluorescent probe 2′,7′-dichlorofluorescin diacetate (DCFH-DA) was employed to detect intracellular ROS levels. Briefly, cells were plated in 6-well plates and incubated overnight. Cells were treated with ESI or cisplatin alone or their combination for the indicated times. Next, the cells were stained with 10 μM DCFH-DA for 30 min before collecting. For quantitative assessment of intracellular ROS levels, the cells were collected and analyzed by FACSCalibur flow cytometer.

### Western Blot Analysis

Cells were seeded in 6-well plates and incubated overnight. After various treatments, the cells were washed once with 1 ml of phosphate-buffered saline and lysed using cell lysis buffer. The same amount of lysate proteins were separated by 10% SDS-PAGE and electroblotted onto PVDF transfer membranes. The blots were blocked with five percent non-fat milk in TBST for 2 h at room temperature. Then incubated with specific primary antibodies overnight at 4°C. HRP-conjugated secondary antibodies and ECL substrate (Bio-Rad, Hercules, CA, United States) were used for detection.

### Measurement of TrxR1 Activity

Cells were seeded in 6-well plates and incubated overnight. Next, the cells were treated with ESI for the indicated time periods and lysed with lysis buffer. TrxR1 activity in cell lysates was measured using an endpoint insulin reduction assay as previously described (Zou et al., 2016). The TrxR1 (14638, Cayman Chemical, MI, United States) activity was determined at room temperature using the DTNB assay. The NADPH-reduced TrxR1 (170 nM) protein was treated with varying concentrations of ESI for the indicated time in a 96-well plates. A master mixture of Tris-EDTA buffer (1 mM EDTA, 50 mM Tris-HCl, pH 7.5) containing NADPH (200 μM) and DTNB (2 mM) was added. The linear increase in absorbance at 412 nm during the initial 3 min was recorded.

### Transient Transfection of Small Interfering RNA (siRNA)

The siRNA duplexes used in this study were obtained from Sigma (St. Louis, MO, United States). The sequences of siRNA were described previously (Zou et al., 2016). Sense 5′-(CUUUGCAGCUGCGCUCAAA)dTdT-3′, antisense 5′-(UUUGAGCGCAGCUGCAAAG)dT dT-3′. The siRNA duplexes targeting TrxR1 were transduced into HCT116 cells. Forty-eight hours post-transduction, the cells were washed with complete media and plated with or without ESI for 24 h for assessing cell survival.

### Docking of ESI to the TrxR1 Structural Model

The crystal structure of rat TrxR1 (PDB code 3EAN, chainA and chain B) was used for present docking study as described previously (Cheng et al., 2009; Liu et al., 2019). The center co-ordination of dock pocket was set as 1.49, 5.74, and 159.58. A grid box size of 60 × 60 × 60 points with a spacing of 0.375 Å between the grid points was implemented. The default parameters were used for running the docking simulation.

### Immunofluorescence Staining

Cells were seeded on sterile cover glasses placed in the 6-well plates and incubated overnight. Next, the cells were treated with ESI or cisplatin alone or their combination for 20 h. For immunofluorescence, the cells were stained with a primary antibody (53BP1, 1:2,000 dilution) overnight at 4°C. Next, the cells were incubated with a DyLight 488 conjugated secondary antibody for 1.5 h at room temperature. The images were obtained using a Leica fluorescence microscope.

### Immunohistochemistry Staining

For immunohistochemistry, 5-μm sections from paraformaldehyde-fixed paraffin-embedded tissues were deparaffinized in xylenes solvent and rehydrated through a graded alcohol series. Immunohistochemistry analyses of Ki-67 and γ-H2A.X were performed according to the protocol described previously (He et al., 2019).

### Xenograft Experiments

Five-week-old athymic BALB/c nude mice (total *n* = 24) were used for *in vivo* experiments. All animals used in this study were handled according to the Institutional Animal Care and Use Committee (IACUC) guidelines, Wenzhou Medical University. The animals were housed at a constant room temperature with a 12 h light/12 h dark cycle and fed a standard rodent diet and water. HCT116 cells (5 × 10^6^ cells in 100 μl of phosphate-buffered saline) were injected subcutaneously into the right back of nude mice. The mice were treated with ESI, cisplatin, or the combination by intraperitoneal (i.p.) injection once every other day at the indicated doses. The tumor volumes were measured to observe dynamic changes in tumor growth and calculated according to the formula: V (mm^3^) = 0.5 × *D* × *d*^2^, where *D* and *d* are the longest and the shortest diameters, respectively. At the end of the experiment, all nude mice were sacrificed, and the tumor tissues were removed and measured.

### MDA Assay

Malondialdehyde is a terminal product of lipid peroxidation. For the MDA assay, tissue proteins of tumor xenograft were homogenized in ice-cold RIPA buffer. The protein concentrations were determined using the Bradford assay (Bio-Rad, Hercules, CA, United States). The MDA levels were detected according to the protocol described previously (Zou et al., 2016).

### Statistical Analysis

The data are expressed as means ± standard error of the mean (SEM). Significant differences between control and experimental groups were determined by *t*-test analyses using statistical software, GraphPad Prism 5.0. A probability (*P*) value of <0.05 was considered statistically significant.

## Data Availability Statement

All datasets presented in this study are included in the article/supplementary material.

## Ethics Statement

The animal study was reviewed and approved by the Institutional Animal Care and Use Committee (IACUC) guidelines, Wenzhou Medical University.

## Author Contributions

YX and PZo designed the study. YX and PZo supervised the study and wrote the manuscript. LH and JC performed the experiments. FaW, FeW, XS, PZh, RS, KL, ZL, DC, GL, and YC collected and analyzed the data. All authors contributed to the article and approved the submitted version.

## Conflict of Interest

The authors declare that the research was conducted in the absence of any commercial or financial relationships that could be construed as a potential conflict of interest.

## Abbreviations

ROS: reactive oxygen species
TrxR1: thioredoxin reductase 1
JNK: c-JunN-terminal kinase
NAC: *N*-acetyl -L-cysteine
DCFH-DA: 2′ ,7′ -dichlorofluorescin diacetate
MDA: malondialdehyde
DTNB: 5,5’-dithiobis(2-nitrobenzoic acid)
CI: combination index.

## References

[B1] Ahmed E. M. Bandopadhyay G. Coyle B. Grabowska A. (2018). A HIF-independent, CD133-mediated mechanism of cisplatin resistance in glioblastoma cells. *Cell Oncol.* 41 319–328. 10.1007/s13402-018-0374-8 29492900 PMC5951876

[B2] Al-Batran S. E. Homann N. Pauligk C. Goetze T. O. Meiler J. Kasper S. (2019). Perioperative chemotherapy with fluorouracil plus leucovorin, oxaliplatin, and docetaxel versus fluorouracil or capecitabine plus cisplatin and epirubicin for locally advanced, resectable gastric or gastro-oesophageal junction adenocarcinoma (FLOT4): a randomised, phase 2/3 trial. *Lancet* 393 1948–1957. 30982686 10.1016/S0140-6736(18)32557-1

[B3] Arner E. S. J. (2017). Targeting the selenoprotein thioredoxin reductase 1 for anticancer therapy. *Adv. Cancer Res.* 136 139–151. 10.1016/bs.acr.2017.07.005 29054416

[B4] Arnold M. Sierra M. S. Laversanne M. Soerjomataram I. Jemal A. Bray F. (2017). Global patterns and trends in colorectal cancer incidence and mortality. *Gut* 66 683–691. 10.1136/gutjnl-2015-310912 26818619

[B5] Basu A. Krishnamurthy S. (2010). Cellular responses to Cisplatin-induced DNA damage. *J. Nucleic Acids* 2010 201367. 10.4061/2010/201367 PMC2929606 20811617

[B6] Bragado P. Armesilla A. Silva A. Porras A. (2007). Apoptosis by cisplatin requires p53 mediated p38alpha MAPK activation through ROS generation. *Apoptosis* 12 1733–1742. 10.1007/s10495-007-0082-8 17505786

[B7] Cheng Q. Sandalova T. Lindqvist Y. Arner E. S. (2009). Crystal structure and catalysis of the selenoprotein thioredoxin reductase 1. *J. Biol. Chem.* 284 3998–4008. 10.1074/jbc.m807068200 19054767

[B8] Chou T. C. (2010). Drug combination studies and their synergy quantification using the Chou-Talalay method. *Cancer Res.* 70 440–446. 10.1158/0008-5472.can-09-1947 20068163

[B9] Cruz-Bermudez A. Laza-Briviesca R. Vicente-Blanco R. J. Garcia-Grande A. Coronado M. J. Laine-Menendez S. (2019). Cisplatin resistance involves a metabolic reprogramming through ROS and PGC-1alpha in NSCLC which can be overcome by OXPHOS inhibition. *Free Radic. Biol. Med.* 135 167–181. 10.1016/j.freeradbiomed.2019.03.009 30880247

[B10] Dagnell M. Schmidt E. E. Arner E. S. J. (2018). The A to Z of modulated cell patterning by mammalian thioredoxin reductases. *Free Radic. Biol. Med.* 115 484–496. 10.1016/j.freeradbiomed.2017.12.029 29278740 PMC5771652

[B11] Dias R. B. De Araujo T. B. S. De Freitas R. D. Rodrigues A. Sousa L. P. Sales C. B. S. (2018). beta-Lapachone and its iodine derivatives cause cell cycle arrest at G2/M phase and reactive oxygen species-mediated apoptosis in human oral squamous cell carcinoma cells. *Free Radic. Biol. Med.* 126 87–100. 10.1016/j.freeradbiomed.2018.07.022 30071298

[B12] Du Y. Zhang H. Lu J. Holmgren A. (2012). Glutathione and glutaredoxin act as a backup of human thioredoxin reductase 1 to reduce thioredoxin 1 preventing cell death by aurothioglucose. *J. Biol. Chem.* 287 38210–38219. 10.1074/jbc.m112.392225 22977247 PMC3488090

[B13] Duan D. Zhang J. Yao J. Liu Y. Fang J. (2016). Targeting thioredoxin reductase by parthenolide contributes to inducing apoptosis of HeLa cells. *J. Biol. Chem.* 291 10021–10031. 10.1074/jbc.m115.700591 27002142 PMC4858956

[B14] Fournel L. Wu Z. Stadler N. Damotte D. Lococo F. Boulle G. (2019). Cisplatin increases PD-L1 expression and optimizes immune check-point blockade in non-small cell lung cancer. *Cancer Lett.* 464 5–14. 10.1016/j.canlet.2019.08.005 31404614

[B15] Glasauer A. Chandel N. S. (2014). Targeting antioxidants for cancer therapy. *Biochem. Pharmacol.* 92 90–101. 10.1016/j.bcp.2014.07.017 25078786

[B16] Gorrini C. Harris I. S. Mak T. W. (2013). Modulation of oxidative stress as an anticancer strategy. *Nat. Rev. Drug Discov.* 12 931–947. 10.1038/nrd4002 24287781

[B17] Harris I. S. Treloar A. E. Inoue S. Sasaki M. Gorrini C. Lee K. C. (2015). Glutathione and thioredoxin antioxidant pathways synergize to drive cancer initiation and progression. *Cancer Cell* 27 211–222. 10.1016/j.ccell.2014.11.019 25620030

[B18] He L. Lai H. Chen T. (2015). Dual-function nanosystem for synergetic cancer chemo-/radiotherapy through ROS-mediated signaling pathways. *Biomaterials* 51 30–42. 10.1016/j.biomaterials.2015.01.063 25770995

[B19] He W. Cao P. Xia Y. Hong L. Zhang T. Shen X. (2019). Potent inhibition of gastric cancer cells by a natural compound via inhibiting TrxR1 activity and activating ROS-mediated p38 MAPK pathway. *Free Radic. Res.* 53 104–114. 10.1080/10715762.2018.1558448 30668191

[B20] Hsu J. H. Chang P. M. Cheng T. S. Kuo Y. L. Wu A. T. Tran T. H. (2019). Identification of Withaferin A as a potential candidate for anti-cancer therapy in non-small cell lung cancer. *Cancers* 11:1003. 10.3390/cancers11071003 31319622 PMC6678286

[B21] Ichikawa H. Nair M. S. Takada Y. Sheeja D. B. Kumar M. A. Oommen O. V. (2006). Isodeoxyelephantopin, a novel sesquiterpene lactone, potentiates apoptosis, inhibits invasion, and abolishes osteoclastogenesis through suppression of nuclear factor-kappaB (nf-kappaB) activation and nf-kappaB-regulated gene expression. *Clin. Cancer Res.* 12 5910–5918. 10.1158/1078-0432.ccr-06-0916 17021000

[B22] Jin R. Gao Y. Zhang S. Teng F. Xu X. Aili A. (2015). Trx1/TrxR1 system regulates post-selected DP thymocytes survival by modulating ASK1-JNK/p38 MAPK activities. *Immunol. Cell Biol.* 93 744–752. 10.1038/icb.2015.36 25753394

[B23] Jung Y. Lippard S. J. (2007). Direct cellular responses to platinum-induced DNA damage. *Chem. Rev.* 107 1387–1407. 10.1021/cr068207j 17455916

[B24] Kabeer F. A. Sreedevi G. B. Nair M. S. Rajalekshmi D. S. Gopalakrishnan L. P. Prathapan R. (2014). Isodeoxyelephantopin from *Elephantopus scaber* (Didancao) induces cell cycle arrest and caspase-3-mediated apoptosis in breast carcinoma T47D cells and lung carcinoma A549 cells. *Chin. Med.* 9:14. 10.1186/1749-8546-9-14 24742378 PMC4003511

[B25] Kengen J. Deglasse J. P. Neveu M. A. Mignion L. Desmet C. Gourgue F. (2018). Biomarkers of tumour redox status in response to modulations of glutathione and thioredoxin antioxidant pathways. *Free Radic. Res.* 52 256–266. 10.1080/10715762.2018.1427236 29320894

[B26] Li Y. Ooi L. S. Wang H. But P. P. Ooi V. E. (2004). Antiviral activities of medicinal herbs traditionally used in southern mainland China. *Phytother. Res.* 18 718–722. 10.1002/ptr.1518 15478204

[B27] Liao W. Wang Z. Fu Z. Ma H. Jiang M. Xu A. (2019). p62/SQSTM1 protects against cisplatin-induced oxidative stress in kidneys by mediating the cross talk between autophagy and the Keap1-Nrf2 signalling pathway. *Free Radic. Res.* 53 800–814. 10.1080/10715762.2019.1635251 31223046

[B28] Lien E. C. Lyssiotis C. A. Juvekar A. Hu H. Asara J. M. Cantley L. C. (2016). Glutathione biosynthesis is a metabolic vulnerability in PI(3)K/Akt-driven breast cancer. *Nat. Cell Biol.* 18 572–578. 10.1038/ncb3341 27088857 PMC4848114

[B29] Liu N. Wang K. S. Qi M. Zhou Y. J. Zeng G. Y. Tao J. (2018). Vitexin compound 1, a novel extraction from a Chinese herb, suppresses melanoma cell growth through DNA damage by increasing ROS levels. *J. Exp. Clin. Cancer Res.* 37:269. 10.1186/s13046-018-0897-x PMC6219156 30400954

[B30] Liu R. Shi D. Zhang J. Li X. Han X. Yao X. (2019). Virtual screening-guided discovery of thioredoxin reductase inhibitors. *Toxicol. Appl. Pharmacol.* 370 106–116. 10.1016/j.taap.2019.03.014 30898620

[B31] Mantzaris M. D. Bellou S. Skiada V. Kitsati N. Fotsis T. Galaris D. (2016). Intracellular labile iron determines H2O2-induced apoptotic signaling via sustained activation of ASK1/JNK-p38 axis. *Free Radic. Biol. Med.* 97 454–465. 10.1016/j.freeradbiomed.2016.07.002 27387771

[B32] Newman D. J. Cragg G. M. (2016). Natural products as sources of new drugs from 1981 to 2014. *J. Nat. Prod.* 79 629–661. 10.1021/acs.jnatprod.5b01055 26852623

[B33] Nuvoli B. Camera E. Mastrofrancesco A. Briganti S. Galati R. (2018). Modulation of reactive oxygen species via ERK and STAT3 dependent signalling are involved in the response of mesothelioma cells to exemestane. *Free Radic. Biol. Med.* 115 266–277. 10.1016/j.freeradbiomed.2017.12.008 29229551

[B34] Pan C. Jin L. Wang X. Li Y. Chun J. Boese A. C. (2019). Inositol-triphosphate 3-kinase B confers cisplatin resistance by regulating NOX4-dependent redox balance. *J. Clin. Invest.* 130 2431–2445. 10.1172/jci124550 31081803 PMC6546469

[B35] Park H. Jeong Y. J. Han N. K. Kim J. S. Lee H. J. (2018). Oridonin enhances radiation-induced cell death by promoting DNA damage in non-small cell lung cancer cells. *Int. J. Mol. Sci.* 19:2378. 10.3390/ijms19082378 30104472 PMC6121891

[B36] Poli A. Nicolau M. Simoes C. M. Nicolau R. M. Zanin M. (1992). Preliminary pharmacologic evaluation of crude whole plant extracts of Elephantopus scaber. Part I: in vivo studies. *J. Ethnopharmacol.* 37 71–76. 10.1016/0378-8741(92)90005-c 1453704

[B37] Rabik C. A. Dolan M. E. (2007). Molecular mechanisms of resistance and toxicity associated with platinating agents. *Cancer Treat. Rev.* 33 9–23. 10.1016/j.ctrv.2006.09.006 17084534 PMC1855222

[B38] Rajesh M. G. Latha M. S. (2001). Hepatoprotection by Elephantopus scaber Linn. *in CCl*4-induced liver injury. *Ind. J. Physiol. Pharmacol.* 45 481–486. 11883157

[B39] Rashmi R. Huang X. Floberg J. M. Elhammali A. E. Mccormick M. L. Patti G. J. (2018). Radioresistant cervical cancers are sensitive to inhibition of glycolysis and redox metabolism. *Cancer Res.* 78 1392–1403. 10.1158/0008-5472.can-17-2367 29339540 PMC5856626

[B40] Roh J. L. Kim E. H. Jang H. Shin D. (2017). Aspirin plus sorafenib potentiates cisplatin cytotoxicity in resistant head and neck cancer cells through xCT inhibition. *Free Radic. Biol. Med.* 104 1–9. 10.1016/j.freeradbiomed.2017.01.002 28057599

[B41] Roy S. Kar M. Saha A. Padhi S. Banerjee B. (2018). Role of beta-catenin in cisplatin resistance, relapse and prognosis of head and neck squamous cell carcinoma. *Cell. Oncol.* 41 185–200. 10.1007/s13402-017-0365-1 29243047 PMC12995243

[B42] Schieber M. Chandel N. S. (2014). ROS function in redox signaling and oxidative stress. *Curr Biol.* 24 R453–R462. 24845678 10.1016/j.cub.2014.03.034 PMC4055301

[B43] Srinivas U. S. Tan B. W. Q. Vellayappan B. A. Jeyasekharan A. D. (2018). ROS and the DNA damage response in cancer. *Redox Biol.* 25:101084. 10.1016/j.redox.2018.101084 30612957 PMC6859528

[B44] Su Y. Yang W. Jiang N. Shi J. Chen L. Zhong G. (2019). Hypoxia-elevated circELP3 contributes to bladder cancer progression and cisplatin resistance. *Int. J. Biol. Sci.* 15 441–452. 10.7150/ijbs.26826 30745833 PMC6367558

[B45] Sun Y. Miao H. Ma S. Zhang L. You C. Tang F. (2018). FePt-Cys nanoparticles induce ROS-dependent cell toxicity, and enhance chemo-radiation sensitivity of NSCLC cells in vivo and in vitro. *Cancer Lett.* 418 27–40. 10.1016/j.canlet.2018.01.024 29331422

[B46] Tang Z. H. Cao W. X. Su M. X. Chen X. Lu J. J. (2017). Osimertinib induces autophagy and apoptosis via reactive oxygen species generation in non-small cell lung cancer cells. *Toxicol. Appl. Pharmacol.* 321 18–26. 10.1016/j.taap.2017.02.017 28237877

[B47] Trachootham D. Alexandre J. Huang P. (2009). Targeting cancer cells by ROS-mediated mechanisms: a radical therapeutic approach? *Nat. Rev. Drug Discov.* 8 579–591. 10.1038/nrd2803 19478820

[B48] Verma S. S. Rai V. Awasthee N. Dhasmana A. Rajalaksmi D. S. Nair M. S. (2019). Isodeoxyelephantopin, a Sesquiterpene lactone induces ROS generation, suppresses NF-kappaB activation, modulates LncRNA expression and exhibit activities against breast cancer. *Sci. Rep.* 9:17980. 10.1038/s41598-019-52971-3 PMC6884568 31784542

[B49] Wang D. Lippard S. J. (2005). Cellular processing of platinum anticancer drugs. *Nat. Rev. Drug Discov.* 4 307–320. 10.1038/nrd1691 15789122

[B50] Wang J. Tian L. Khan M. N. Zhang L. Chen Q. Zhao Y. (2018). Ginsenoside Rg3 sensitizes hypoxic lung cancer cells to cisplatin via blocking of NF-kappaB mediated epithelial-mesenchymal transition and stemness. *Cancer Lett.* 415 73–85. 10.1016/j.canlet.2017.11.037 29199005

[B51] Wang Y. Hao F. Nan Y. Qu L. Na W. Jia C. (2018). PKM2 inhibitor Shikonin overcomes the Cisplatin resistance in bladder cancer by inducing necroptosis. *Int. J. Biol. Sci.* 14 1883–1891. 10.7150/ijbs.27854 30443191 PMC6231221

[B52] Wang Y. Zhang J. Huang Z. H. Huang X. H. Zheng W. B. Yin X. F. (2017). Isodeoxyelephantopin induces protective autophagy in lung cancer cells via Nrf2-p62-keap1 feedback loop. *Cell Death Dis* 8:e2876. 10.1038/cddis.2017.265 28617433 PMC5584574

[B53] Xu J. Cheng Q. Arner E. S. (2016). Details in the catalytic mechanism of mammalian thioredoxin reductase 1 revealed using point mutations and juglone-coupled enzyme activities. *Free Radic. Biol. Med.* 94 110–120. 10.1016/j.freeradbiomed.2016.02.013 26898501

[B54] Yan G. R. Tan Z. Wang Y. Xu M. L. Yu G. Li Y. (2013). Quantitative proteomics characterization on the antitumor effects of isodeoxyelephantopin against nasopharyngeal carcinoma. *Proteomics* 13 3222–3232. 10.1002/pmic.201300152 23970500

[B55] Yang C. Lim W. Bazer F. W. Song G. (2017). Myricetin suppresses invasion and promotes cell death in human placental choriocarcinoma cells through induction of oxidative stress. *Cancer Lett.* 399 10–19. 28428076 10.1016/j.canlet.2017.04.014

[B56] Yao J. Duan D. Song Z. L. Zhang J. Fang J. (2020). Sanguinarine as a new chemical entity of thioredoxin reductase inhibitor to elicit oxidative stress and promote tumor cell apoptosis. *Free Radic. Biol. Med.* 152 659–667. 10.1016/j.freeradbiomed.2020.01.008 31931095

[B57] Zhang T. Zheng P. Shen X. Shao R. Wang B. Shen H. (2019). Curcuminoid WZ26, a TrxR1 inhibitor, effectively inhibits colon cancer cell growth and enhances cisplatin-induced cell death through the induction of ROS. *Free Radic. Biol. Med.* 141 93–102. 10.1016/j.freeradbiomed.2019.06.005 31176737

[B58] Zhao C. Zhang Y. Zou P. Wang J. He W. Shi D. (2015). Synthesis and biological evaluation of a novel class of curcumin analogs as anti-inflammatory agents for prevention and treatment of sepsis in mouse model. *Drug Des. Devel. Ther.* 9 1663–1678. 10.2147/dddt.s75862 25834403 PMC4370917

[B59] Zheng X. Chen Y. Bai M. Liu Y. Xu B. Sun R. (2019). The antimetastatic effect and underlying mechanisms of thioredoxin reductase inhibitor ethaselen. *Free Radic. Biol. Med.* 131 7–17. 10.1016/j.freeradbiomed.2018.11.030 30496814

[B60] Zou P. Xia Y. Ji J. Chen W. Zhang J. Chen X. (2016). Piperlongumine as a direct TrxR1 inhibitor with suppressive activity against gastric cancer. *Cancer Lett.* 375 114–126. 10.1016/j.canlet.2016.02.058 26963494

